# **β**-Thalassemia: New Therapeutic Modalities, Genetics, Complications, and Quality of Life

**DOI:** 10.1155/2012/902067

**Published:** 2012-09-18

**Authors:** Mehran Karimi, Sezaneh Haghpanah, Ali T. Taher, Maria Domenica Cappellini

**Affiliations:** ^1^Hematology Research Center, Shiraz University of Medical Sciences, Shiraz, Iran; ^2^American University of Beirut Medical Center, Beirut, Lebanon; ^3^IRCCS Ca' Granda Foundation Maggiore Policlinico Hospital, University of Milan, Milan, Italy

Beta-thalassemia is considered one of the most common genetic disorders which mass migration is introducing to countries worldwide and challenging them with its management. Advanced and improved medical care has allowed us to prolong the lives of thalassemia patients, simultaneously uncovering novel complications and outlining new challenges. We herein present in this special issue of *Anemia* some of the most cutting-edge research outcomes pertaining to the latest complications and the novel treatment strategies in iron-chelation therapy. We also study the effects of these interventions on the quality of life of patients. It is studies like these that serve as the basis of evidence-based medicine and guide clinicians through their process of decision making. 

One article of this special issue addresses the new method of using transactional Doppler ultrasonography for measuring intracranial blood flow velocity in patients with beta-thalassemia intermedia. This study shows higher blood flow velocity in these patients compared to the control group, which may point to a higher risk of ischemic events in the future. 

Another paper discusses that thalassemic DNA-containing red blood cells are under oxidative stress, which induces externalization of phosphatidylserine. This mechanism is involved in the removal of these cells from the circulation by the spleen, similar to that of the removal of senescent red blood cells.

This special issue also includes a paper explaining the pathophysiology of bone modifications in beta-thalassemia. Imbalance in mineral turnover resulting in abnormal regulation of bone metabolism may be related to hormonal and genetic factors, iron overload, and iron chelation therapy. These factors and their contribution are addressed.

Moreover, a review article that addresses the mechanism of tissue damage arising from iron overload in patients with *β*-thalassemia major is presented. It is the result of oxidative stress from free radical production, altered antioxidant enzymes, and its interaction with other essential trace element levels.

The last paper presented is a study comparing the quality of life in patients with *β*-thalassemia major and myelodysplastic syndrome with iron-overload treated either with deferasirox or deferoxamine injections. The results show that deferasirox can improve health-related quality of life treatment satisfaction and adherence compared to subcutaneous deferoxamine injection. This issue is crucial and often neglected in the long-term treatment of patients with iron overload.

*Mehran Karimi* *Sezaneh Haghpanah* *Ali T. Taher* *Maria Domenica Cappellini*

